# Radioimmunotherapy as a pathogen-agnostic treatment method for opportunistic mucormycosis infections

**DOI:** 10.1099/acmi.0.000671.v4

**Published:** 2023-12-06

**Authors:** Jorge L.C. Carvalho, Mackenzie E. Malo, Kevin J.H. Allen, Connor Frank, Zhiwen Xiao, Rubin Jiao, Ekaterina Dadachova

**Affiliations:** ^1^​ College of Pharmacy and Nutrition, University of Saskatchewan, Saskatoon, Saskatchewan S7N 5E5, Canada

**Keywords:** 1, 3-beta-glucan, COVID-19, immunosuppressed, lutetium-177, melanin, mucormycosis, pathogen-agnostic targeting, pan-antigens, radioimmunotherapy, *Rhizopus oryzae*

## Abstract

Invasive fungal infections (IFIs) such as mucormycosis are causing devastating morbidity and mortality in immunocompromised patients as anti-fungal agents do not work in the setting of a suppressed immune system. The coronavirus disease 2019 (COVID-19) pandemic has created a novel landscape for IFIs in post-pandemic patients, resulting from severe immune suppression caused by COVID-19 infection, comorbidities (diabetes, obesity) and immunosuppressive treatments such as steroids. The antigen–antibody interaction has been employed in radioimmunotherapy (RIT) to deliver lethal doses of ionizing radiation emitted by radionuclides to targeted cells and has demonstrated efficacy in several cancers. One of the advantages of RIT is its independence of the immune status of a host, which is crucial for immunosuppressed post-COVID-19 patients. In the present work we targeted the fungal pan-antigens 1,3-beta-glucan and melanin pigment, which are present in the majority of pathogenic fungi, with RIT, thus making such targeting pathogen-agnostic. We demonstrated in experimental murine mucormycosis in immunocompetent and immunocompromised mice that lutetium-177 (^177^Lu)-labelled antibodies to these two antigens effectively decreased the fungal burden in major organs, including the brain. These results are encouraging because they show the effectiveness of pathogen-agnostic RIT in significantly decreasing fungal burden *in vivo*, while they can also potentially be applied to treat the broad range of invasive fungal infections that express the pan-antigens 1,3-beta-glucan or melanin.

## Data availability statement

All data are available in the article and Supplementary Material.

## Introduction

Invasive fungal infections (IFIs) are very common not only in hospitalized patients, but also in immunocompromised individuals, organ transplant recipients, uncontrolled diabetics and HIV/AIDS patients – the proportion of IFIs is roughly 6 cases per 100 000 individuals/year [1]. According to [1], ‘only half of such infections are detected during the patient’s lifetime, making this one of the more common overlooked causes of death in intensive-care patients’. Mucormycosisis is an IFI caused by different mycomycetes, including *Rhizopus* species, with some countries, e.g. India, having a relatively high mucormycosis prevalence of 14 cases per 100 000 individuals/year [2]. Mucormycosis-causing fungi are omnipresent in nature and are found in the soil, animal excrement, rotting vegetables and other biological material [3]. Before coronavirus disease 2019 (COVID-19), species of *Rhizopus* lead to a high incidence of and high mortality rate from fungal pneumonias in patients who are immunocompromised or completely immunosuppressed due to haematological malignancies and haematological stem cell transplants [4]. The COVID-19 pandemic created a novel landscape for opportunistic fungal infections, including mucormycosis, in post-COVID-19 patients whose immunosuppression results from a combination of factors: severe immune dysfunction caused by COVID-19 disease, COVID-19 treatment with high doses of steroids to reduce inflammation, and comorbidities (with diabetes being the most prominent) [5–7]. Due to severe immune suppression, standard anti-fungal treatments [8, 9] do not work in such individuals, and novel approaches to treatment that are independent of a patient’s immune status are urgently needed.

Radioimmunotherapy (RIT) utilizes antigen–antibody interaction to deliver lethal doses of ionizing radiation emitted by radionuclides to the targeted cells and has demonstrated efficacy in several types of cancer [10]. Several years ago, we proposed to utilize antibodies for pathogen-agnostic targeting of surface-expressed antigens shared by major opportunistic fungal pathogens (so called pan-antigens) such as melanin, heat shock protein 60 (HSP60) and beta-glucans [11]. These antigens are exposed on the surface of fungal cells and thus are accessible to the radiolabelled mAbs for binding and delivering cytocidal payloads to those cells. Using *Cryptococcus neoformans* and *Candida albicans* as model organisms *in vitro* and *Blastomyceses dermatitidis in vivo* we showed that mAbs to 1,3-beta-glucan and melanin pan-antigens killed 80–100 % of fungal cells when radiolabelled with alpha-particles emitting radionuclides [12, 13]. The previously demonstrated safety of anti-fungal RIT in murine models was confirmed by our recent safety study in beagle dogs [14]. In the current work we tested our hypothesis that RIT with a beta-emitting radionuclide lutetium-177 (^177^Lu) targeting fungal pan-antigens 1,3-beta-glucan and melanin expressed by *Rhizopus oryzae* can be effective in treating this infection in immunocompetent and immunocompromised murine models.

## Methods

### Antibodies, reagents and radionuclides

Murine IgG1 Isotype Control MOPC21 (MA1-10407) was purchased from Invitrogen (Waltham, MA, USA). Murine anti-(1-3)-beta-glucan antibody (400-2) was purchased from Biosupplies Australia Pty Ltd (Parkville, Australia), murine anti-CTLA4 antibody from InvivoGen (San Diego, CA, USA), humanized anti-CD33 antibody lintuzumab biosimilar from Creative Biolabs (Shirley, NY, USA). Chimeric anti-melanin c8C3 antibody was a gift from Radimmune Therapeutics (Burlingame, CA, USA). The bifunctional chelator S-2-(4-isothiocyanatobenzyl)−1,4,7,10-tetraazacycododecane tetraacetic acid (DOTA) was purchased from Macrocyclics (Dallas, TX, USA). Indium-111 (^111^In) was obtained from BWXT (Cambridge, ON, Canada) and ^177^Lu from McMasters University (Hamilton, ON, Canada). Laminarin from *Laminaria digitata* (cat. #L9634) and melanin from *Sepia officinalis* (cat. #M2649) were procured from Sigma-Aldrich (St Louis, MO, USA).

### *R. oryzae* culture

*R. oryzae* Went et Prinsen Geerligs (cat. # 56 536, ATCC, Manassas, VA, USA), strain designation CBS 112.07 (NRRL 3133, VKM F-1414), was maintained in potato dextrose broth (PDB; 2 % w/v dextrose, 0.4 % w/v potato extract) at 30–35 °C, with subculturing monthly. Sporulation was induced by plating on solid SAB media.

### *In vitro* binding assays

The immunoreactivity of 400–2 mAb towards (1-3)-beta-glucan was tested by laminarin enzyme-linked immunosorbent assay (ELISA) [15, 16] and the immunoreactivity of c8C3 mAb towards melanin was tested by in-house melanin ELISA. These ELISA protocols were subsequently modified to assess the binding of 400–2 and c8C3 mAbs to *R. oryzae* spores and hyphae (File S1, available with the online version of this article).

### Murine *R. oryzae* infection model in immunocompetent mice

**Fig. 1.**
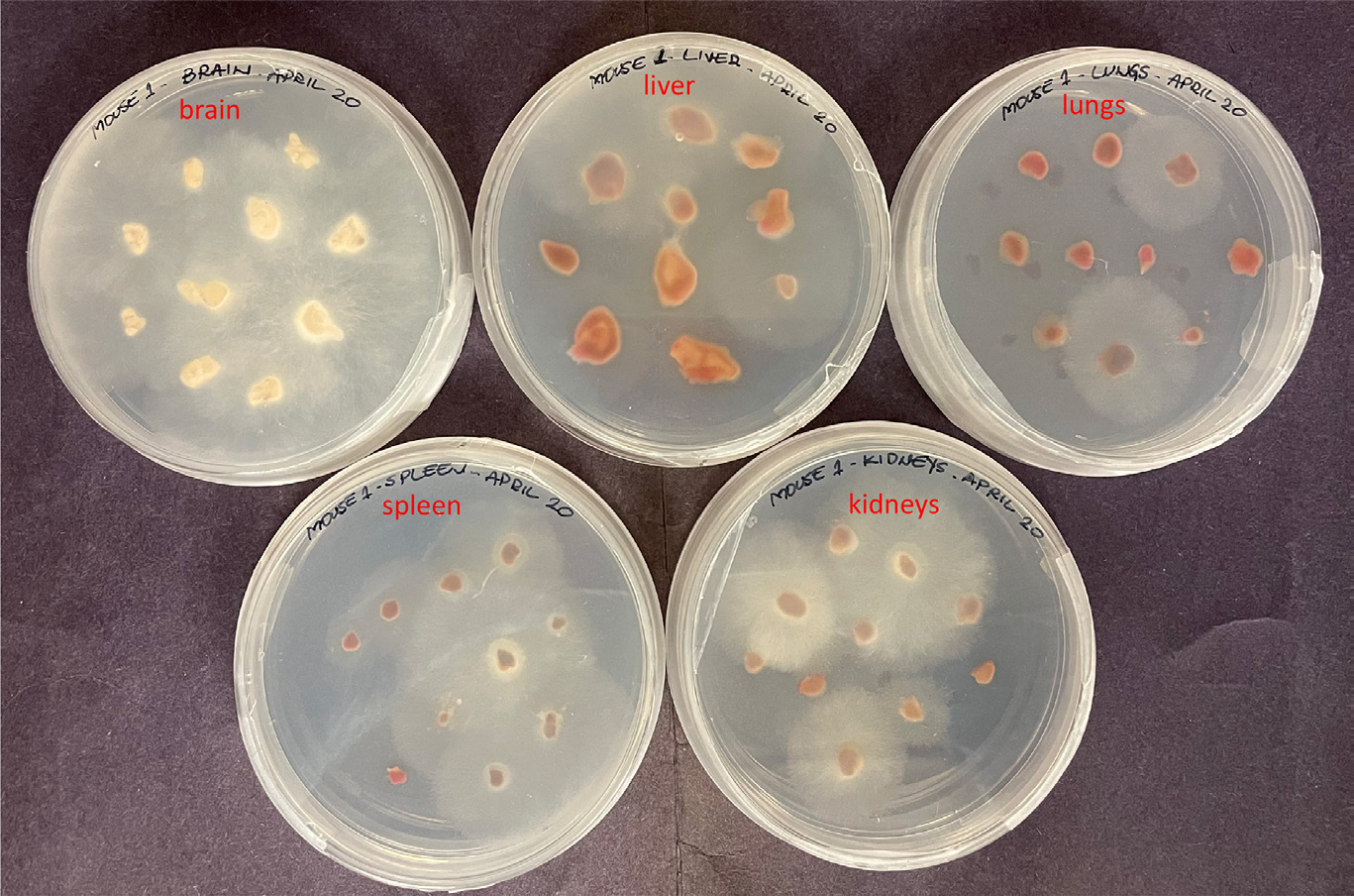
PDA plates showing the fungal growth in the organ of a mouse infected IV with *R. oryzae* and sacrificed 72 h later. From left to right: brain, liver, spleen, lungs and kidneys were divided into 10 fragments and plated.

All animal experiments were conducted in accordance with the humane principles for animal research approved by the Canadian Council on Animal Care (CCAC) and by the Animal Research Ethics Board (AREB) at the University of Saskatchewan, protocol #20 210 106. *R. oryzae* fungal spores were harvested from solid SAB plates, washed twice with phosphate-buffered saline (PBS) and then passed through 70 µm filters to remove remnant hyphae [17]. Female 6–8-week-old C57Bl/6 mice (Charles River Laboratories) were administered 100 µl *R*. *oryzae* spores suspension (3×10^5^ spores ml^−1^) through tail vein injections to induce infection. To assess the organs’ fungal burden, the mice were sacrificed at 72 h post-infection and their organs (brain, liver, spleen, lungs and kidneys) were harvested, divided in into 10 fragments (approximately 2 mm×2 mm) and cultured on PDA plates at 12 °C. Fungal growth on the fragments was measured (colony diameter, cm) three times a day during 148 h after plating (Fig. 1).

### Murine *R. oryzae* infection model in immunocompromised mice

To evaluate whether RIT can be effective in immunocompromised mice, which would recapitulate in some way the immunocompromised patients, we irradiated C57Bl6 female mice with 250 or 500 cGy of X-rays. Whole-body irradiation of rodents is a widely used research tool in immunology and oncology to render rodents reversibly or irreversibly immunosuppressed [18]. Ten female 6–8-week-old C57Bl6 mice were irradiated in a X-RAD Biological Irradiator (North Brandford, CT, USA) using the following parameters: set KV, 225.0; set mA, 13.30; filter, 2.5 mm Al, 0.1 mm Cu; set dose, 250 or 500 cGy; SSD, 50/0.0; exposure size, 29 cm diameter; total time irradiation time, 135 or 270 s, respectively. The irradiation was followed by the assessment of their body weight, white blood cells (WBCs), red blood cells (RBCs) and platelets for 29 days. Based on the results of these experiments, we chose to irradiate C57Bl6 mice with 250 cGy and infect them with *R.oryzae* on day 3 post-irradiation.

### Conjugation and radiolabelling of mAbs

MOPC21, c8C3 and 400–2 mAbs were conjugated to the bifunctional chelating agent DOTA (Macrocyclics, San Antonio, TX, USA) as previously described [13, 18]. Chelating agent to antibody ratios (CARs) for the resulting antibody conjugates were determined via matrix-assisted laser desorption/ionization time-of-flight mass spectrometry (MALDI-TOF MS) at the University of Alberta, Canada, mass spectrometry facility and found to be ~3.1 DOTA/mAb for 400.2-DOTA and ~10.9 DOTA/mAb for c8C3-DOTA. Following conjugation, the mAbs were radiolabelled with ^111^In or ^177^Lu [13, 19]. The radiochemical purity of the radiolabelled mAbs was checked using instant thin-layer chromatography (iTLC) (Agilent Technologies, CA, USA). iTLC strips were developed in 0.15 mM ammonium acetate with a radiolabelled mAb remaining at the application point (Rf=0) while EDTA/DTPA–radionuclide complex was travelling with the solvent front (Rf=1) and subsequently cut in halves and counted in a 2470 Wizard2 Gamma counter (Perkin Elmer, MA, USA). Further quality control of radiolabelled mAbs was performed using radioHPLC (Agilent Technologies, CA, USA). The radioHPLC system was outfitted with a TSKgel G3000SW size-exclusion column, which was isocratically eluted at a flow rate of 0.35 ml min^−1^. When the radiochemical purity of labelled mAbs was <99 %, they were purified via spin filtration using 30 kDa molecular weight cutoff spin filters. Radiochemical purity was confirmed to be >99 % prior to administering radiolabelled mAbs to mice for imaging or therapy.

### Single-photon computer tomography (SPECT)/computer tomography (CT) imaging

Female immunocompetent C57Bl/6 mice were infected with *R. oryzae* spores as above and 24 h post-infection were injected IV via tail vein with ^111^In-labelled 400–2, c8C3 and MOPC21 mAbs (three animals per group) for SPECT/CT on a MILabs VECTor^4^ camera (Netherlands). Images were collected at 24, 48 and 72 h post-antibody administration for 15 min with a XUHS-M collimator 20–350 keV and reconstructed using both 171 and 245 keV ^111^In gamma emissions with a voxel size of 0.4 mm, 10 subsets and 10 iterations.

### RIT of *R. oryzae*-infected immunocompetent and immunocompromised mice with ^177^Lu-labelled 400-2 and c8C3 mAbs

The first series of experiments was conducted in immunocompetent mice using 60 female 6–8-week-old C57Bl/6 mice. Twenty-four hours post-infection, the mice were randomized into the groups of 10 and administered via tail vein injection with the following treatments: (1) left untreated; (2) 50 µCi ^177^Lu–DOTA-400–2; (3) 100 µCi ^177^Lu–DOTA-400–2; (4) 50 µCi ^177^Lu–DOTA–c8C3; (5) 100 µCi ^177^Lu–DOTA–c8C3; (6) 100 µCi ^177^Lu–DOTA–MOPC21. Forty-eight hours post-RIT the animals were sacrificed and tissues (spleen, kidneys, liver, lungs, and brain) were harvested and plated on PDA plates and then fungal growth enumerated as described above. In the second series of experiments 30 female 6–8-week-old C57Bl/6 mice were irradiated with 250 cGy X-rays as described above, and on day 3 after irradiation they were infected IV with 3×10^4^ *R. oryzae* spores. Twenty four hours post-infection they were treated as follows: (1) left untreated; (2) cold (unlabelled) c8C3 mAb; (3) cold 400–2 mAb; (4) 100 µCi ^177^Lu–DOTA-400–2; (5) 100 µCi ^177^Lu–DOTA–c8C3; (6) 100 µCi ^177^Lu–DOTA–MOPC21. Forty eight hours post-RIT the animals were sacrificed and their tissues were harvested and processed for fungal growth as above.

### Statistical analysis

All data were analysed using GraphPad Prism 9.0 software (San Diego, CA, USA). Data are presented as mean±standard deviation (sd). The two-way analysis of variance (ANOVA) test was employed to determine significant differences between the distinct groups and *P* <0.05 was considered statistically significant.

## Results

### mAbs to melanin and 1,3-beta-glucan bound specifically to melanin and 1,3-beta-glucan in *R. oryzae* spores and hyphea

**Fig. 2.**
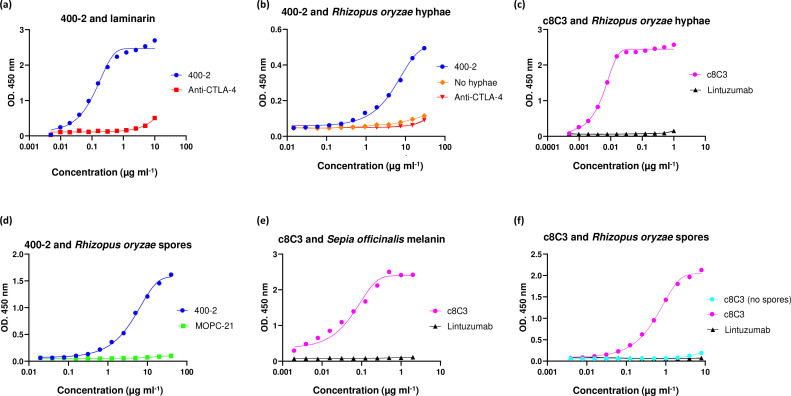
Immunoreactivity of 400-2 and c8C3 mAbs to 1,3-beta-glucan and melanin, respectively, in *R. oryzae* hyphae or spores. (a) Binding of 400-2 mAb to laminarin (1,3-beta-glucan surrogate). (b) Binding of 400-2 mAb to *R. oryzae* hyphae. (c) Binding of 400-2 mAb to *R. oryzae* spores. (d) Binding of c8C3 mAb to *Sepia officinalis* melanin. (e) Binding of c8C3 mAb to *R. oryzae* spores. (f) Binding of c8C3 mAb to *R. oryzae* hyphae. Murine anti-CTLA4 and MOPC21 mAbs were used as negative controls for 400-2, and humanized lintuzumab was used as a negative control for c8C3.

Fig. 2a displays ELISA of mAb 400–2 specifically binding to laminarin, which is used in ELISA format as a surrogate for 1,3-beta-glucan, while c8C3 mAb to melanin bound specifically to its melanin antigen from *S. officinalis* (Fig. 2d). 400–2 mAb was also able to recognize and bind to 1,3-beta-glucan in *R.oryzae* spores and hyphae (Fig. 2b, c) and c8C3 mAb recognized and bound to melanin in *R. oryzae* spores and hyphae (Fig. 2e, f). While the binding of c8C3 was approximately the same to the spores and the hyphae, binding of 400–2 mAb to the hyphae was lower than to the spores.

### mAbs to melanin and 1,3-beta-glucan labelled quantitatively with ^111^In and ^177^Lu

**Fig. 3.**
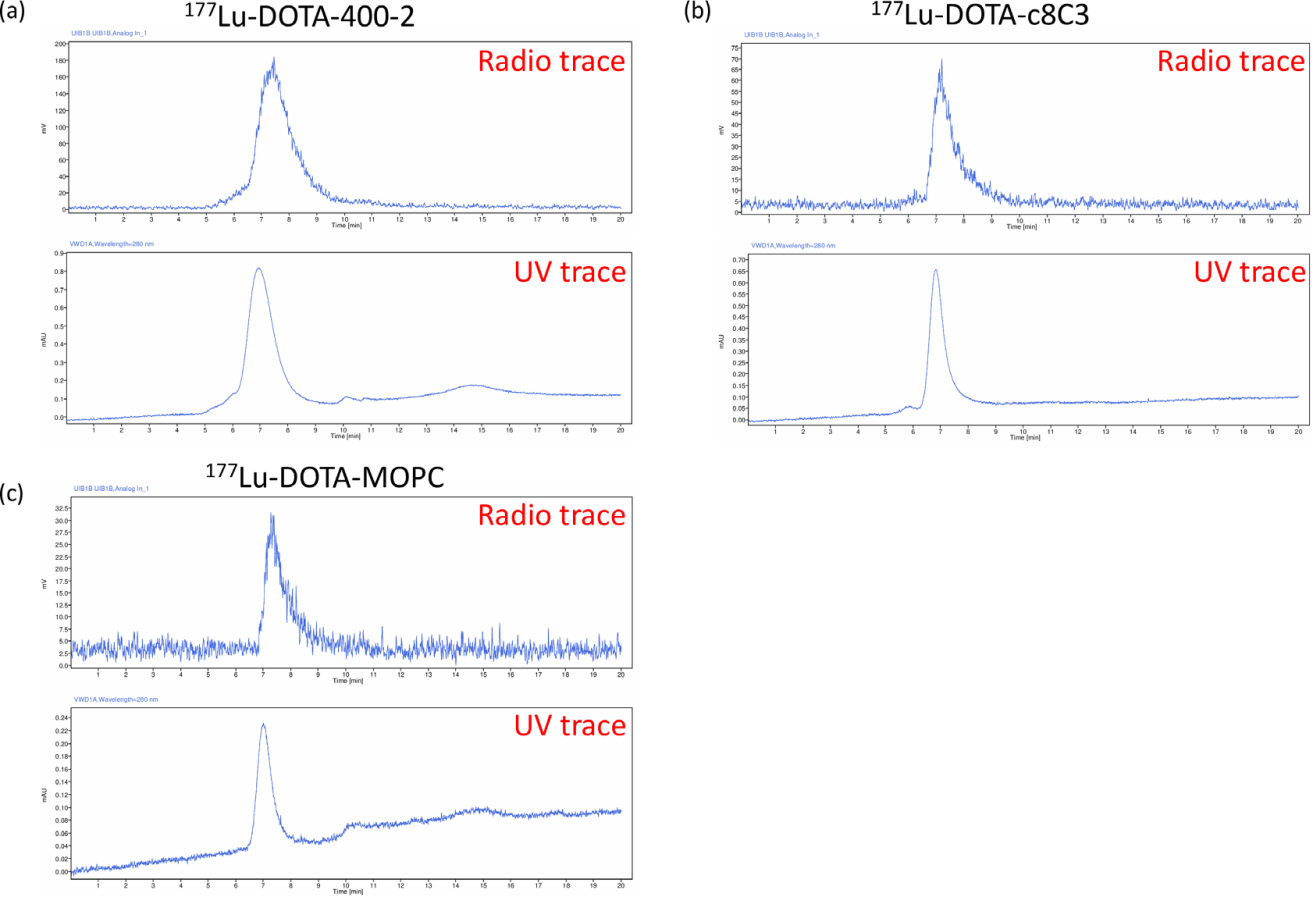
radioHPLC for ^177^Lu-labelled mAbs: ^177^Lu–DOTA-400–2 (**a**), ^177^Lu–DOTA–c8C3 (**b**) and ^177^Lu–DOTA–MOPC21 (**c**). The upper chromatogram is a radioactivity trace and the lower one is a UV trace at 280 nm.

MAbs 400–2 and c8C3 as well as control mAb MOPC21 were labelled quantitatively with ^111^In and ^177^Lu. Fig. 3 shows the radiochromatograms for ^177^Lu–DOTA-400–2 (**a**), ^177^Lu–DOTA–c8C3 (**b**) and ^177^Lu–DOTA–MOPC21 (**c**). This quantitative labelling provided impetus for *in vivo* imaging and RIT experiments in mice with experimental mucormycosis.

### mSPECT/CT demonstrated different biodistribution of fungal antigens-specific and control mAbs

**Fig. 4.**
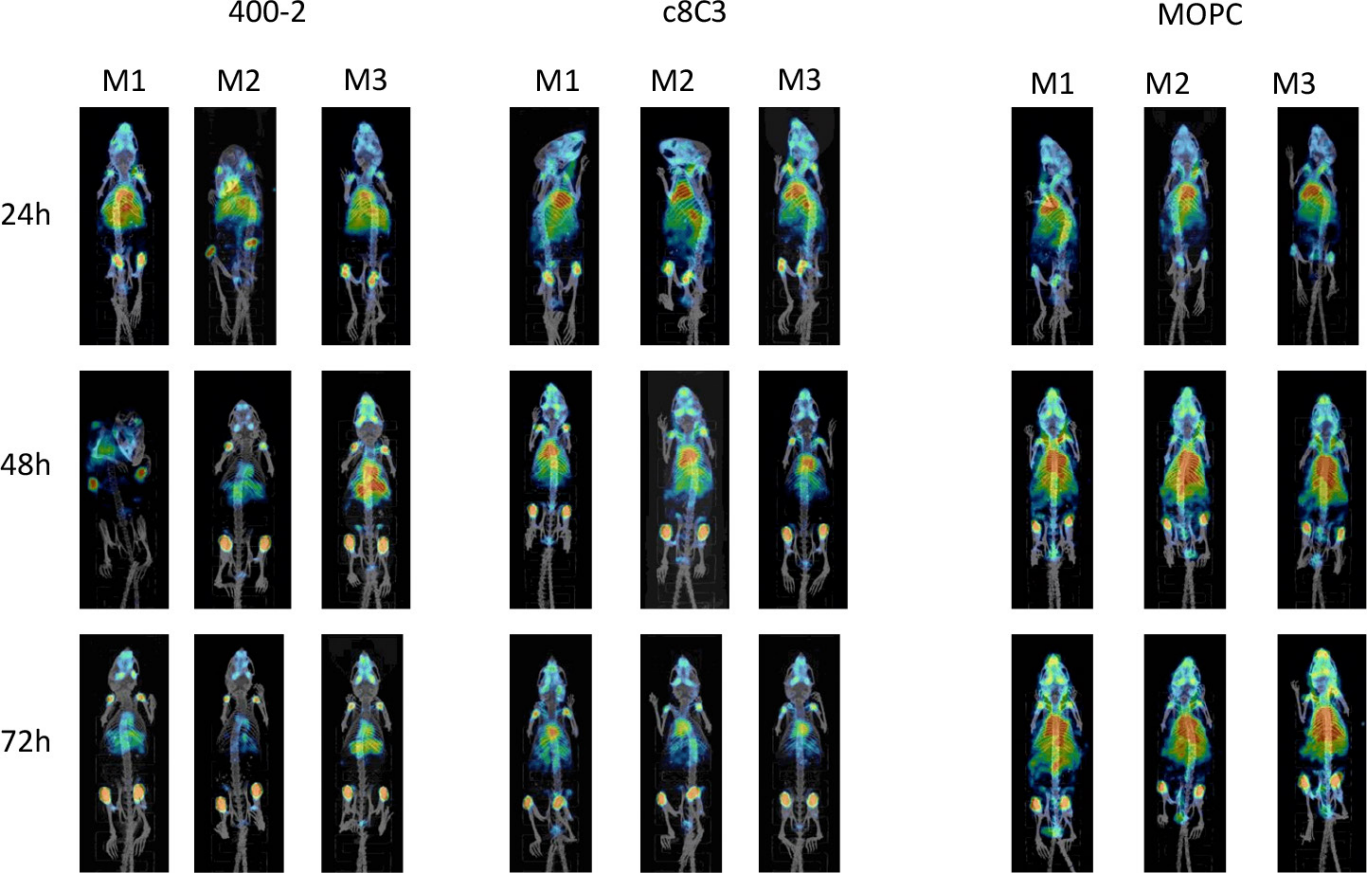
microSPECT/CT of *R. oryzae*-infected C57BL6 mice. Infected mice were injected with 100 µCi of ^111^In–DOTA-400–2 (left panel), ^111^In–DOTA–c8C3 (middle panel) and ^111^In–DOTA–MOPC21 (right panel) and imaged at 24, 48 and 72 h post-administration of the radiolabelled mAbs. M1, M2 and M3 denote animals 1, 2 and 3 in each group. Red colour corresponds to the highest levels of radioactivity in the tissue and blue colour to the lowest. Antibodies are distributed primarily in the thoracic area and in the liver, with some uptake in knee joints due to binding to Fc receptors on the macrophages.

The microSPECT/CT of *R.oryzae*-infected mice revealed biodistribution patterns of fungal antigen-specific mAbs ^111^In-DOTA–c8C3 and ^111^In-DOTA-400–2 were quite different from those of the control mAb ^111^In-DOTA–MOPC21 (Fig. 4). While at 24 h post-administration all three mAbs remained in circulation, starting from 48 h post-administration both ^111^In-DOTA–c8C3 and ^111^In-DOTA-400–2 cleared from the circulation much faster than ^111^In-DOTA–MOPC21, with the former showing the uptake in the lungs, liver and spleen. This trend continued at 72 h post-administration.

### RIT of *R. oryzae*-infected immunocompetent mice reduced the organ fungal burden in a dose-dependent manner

**Fig. 5.**
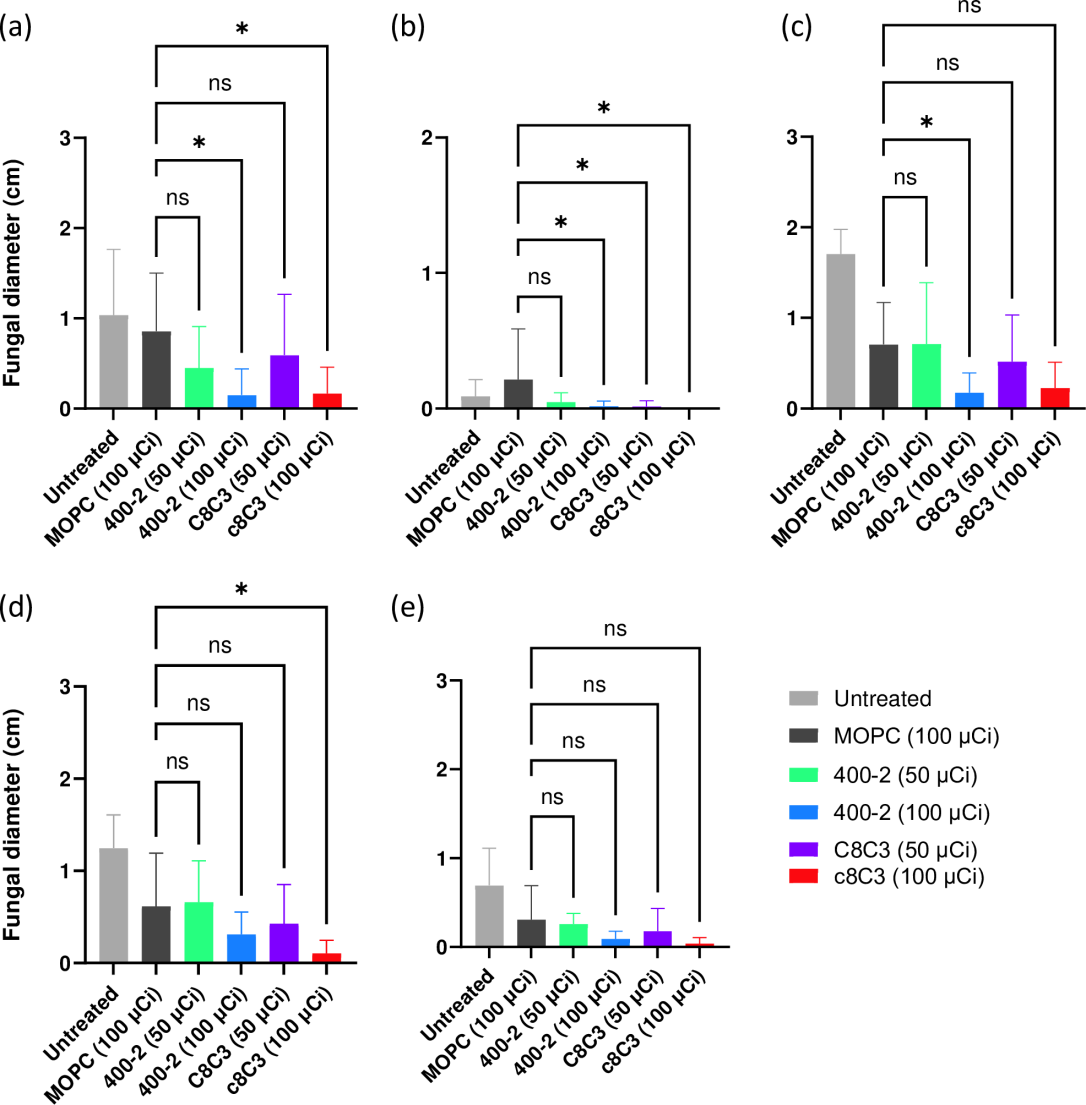
Organ fungal burden of *R. oryzae*-infected immunocompetent C57Bl6 female mice after RIT with ^177^Lu–DOTA-400–2 and ^177^Lu–DOTA–c8C3 mAbs. (**a**) Brain, (**b**) lungs, (**c**) liver, (**d**) spleen and (**e**) kidneys. C57Bl/6 *R*. *oryzae*-infected mice (10 animals per group) were treated IV 24 h post-infection with: 50 µCi ^177^Lu–DOTA-400–2; 100 µCi ^177^Lu–DOTA-400–2; 50 µCi ^177^Lu–DOTA–c8C3; 100 µCi ^177^Lu–DOTA–c8C3; 100 µCi ^177^Lu–DOTA–MOPC21; or left untreated. Forty-eight hours post-RIT the animals were sacrificed and tissues (spleen, kidneys, liver, lungs and brain) were harvested and plated on PDA plates and fungal growth was enumerated. *, *P*<0.01; ns, not significant.

In the first series of RIT experiments we performed RIT of *R.oryzae* in immunocompetent mice. RIT with ^177^Lu–DOTA-400–2 and ^177^Lu–DOTA–c8C3 mAbs resulted in a decrease in fungal burden in all tested organs (Fig. 5). Both mAbs decreased the fungal burden in a dose-dependent manner with 100 µCi being more effective than 50 µCi. The fungus killing effect was clearly antigen-specific when killing with 100 µCi ^177^Lu–DOTA-400–2 and ^177^Lu–DOTA–c8C3 was compared to that of 100 µCi of control ^177^Lu–DOTA–MOPC21 mAb. Overall, radiolabelled c8C3 mAb to melanin and 400–2 mAb to 1,3-beta-glucan proved to be equally effective in reducing the *R. oryzae* fungal burden in immunocompetent mice.

### RIT reduced fungal burden in the organs of *R. oryzae*-infected immunocompromised mice

**Fig. 6.**
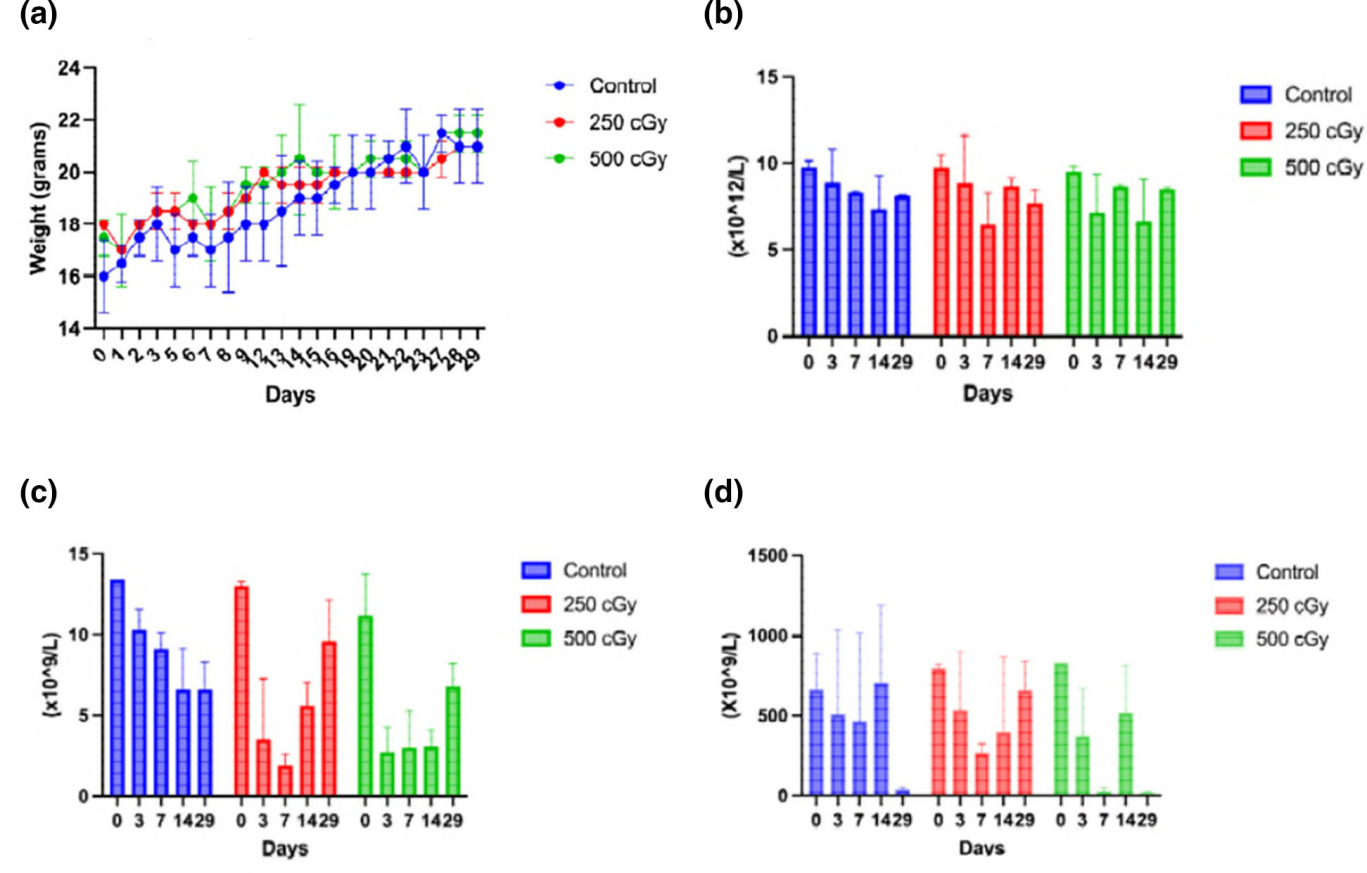
Evaluation of X-ray irradiation effects on C57Bl6 female mice. Mice were irradiated with 250 or 500 cGy X-rays or left untreated (controls) and followed for 29 days: (a) body weights; (**b**) red blood cells; (**c**) white blood cells; (**d**) platelets.

To evaluate whether RIT can be effective in immunocompromised mice, which would recapitulate in some way immunocompromised patients, we irradiated C57Bl6 female mice with 250 or 500 cGy of X-rays. There was no difference in body weights of mice between irradiated and control groups (Fig. 6a) or in the numbers of RBCs (Fig. 6b). The WBC and platelet counts decreased significantly on days 3 and 7 post-irradiation in both 250 and 500 cGy groups, and started to recover on day 14 (Fig. 6c, d). Based on these results, we chose to infect mice with *R. oryzae* on day 3 post-irradiation with 250 cGy.

**Fig. 7.**
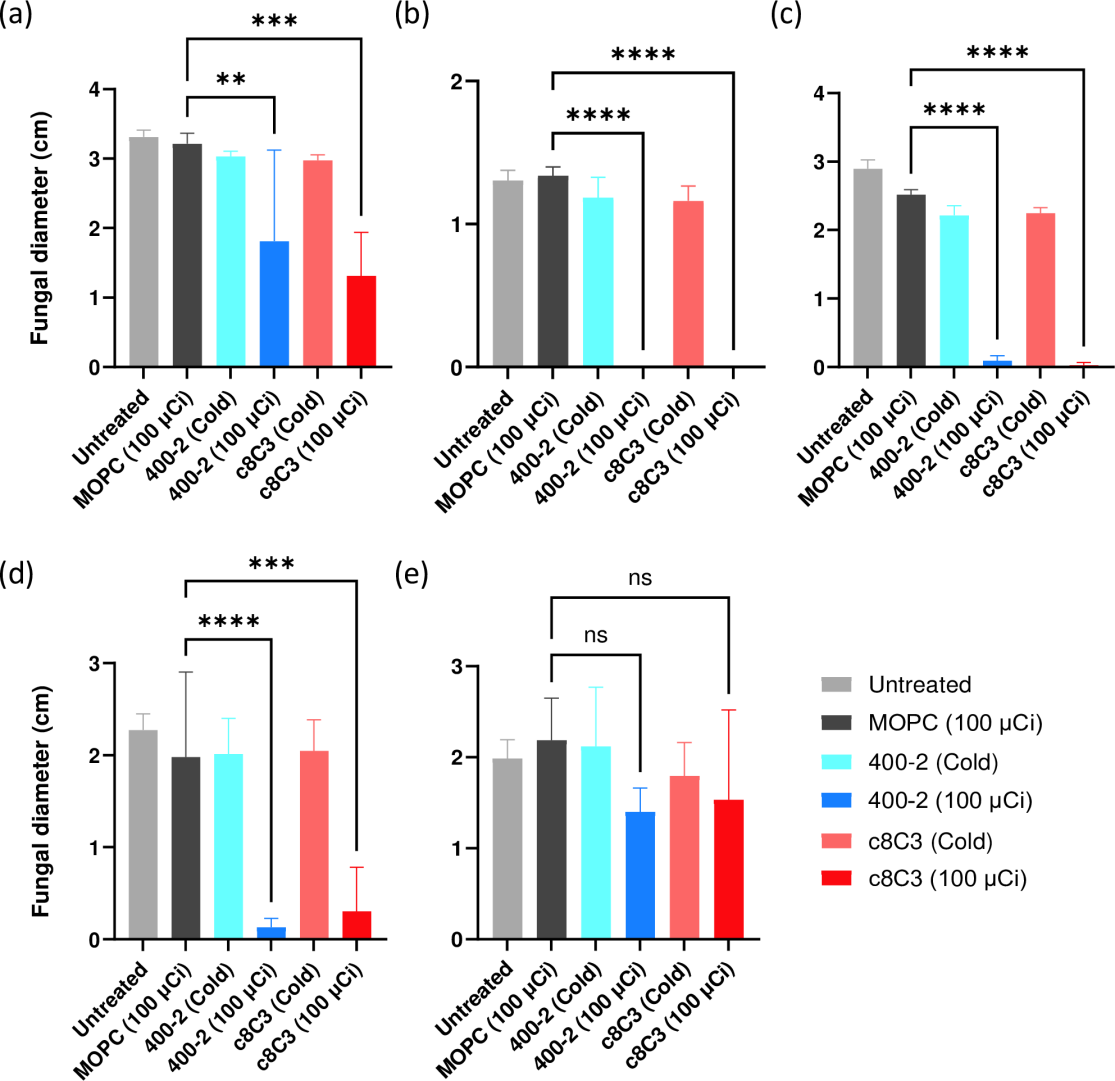
Organ fungal burden of *R. oryzae*-infected immunocompromised C57Bl6 female mice after RIT with ^177^Lu–DOTA-400-2 and ^177^Lu–DOTA–c8C3 mAbs. (a) Brain, (b) lungs, (c) liver, (d) spleen and (e) kidneys. C57Bl/6 mice (five animals per group) were irradiated with 250 cGy X-ray, infected with *R. oryzae* on day 3 post-irradiation and treated IV 24 h post-infection with: 100 µCi ^177^Lu–DOTA-400-2; 100 µCi ^177^Lu–DOTA–c8C3; 100 µCi ^177^Lu–DOTA–MOPC21; cold c8C3; cold 400-2; or left untreated. Forty-eight hours post-RIT the animals were sacrificed and tissues (spleen, kidneys, liver, lungs and brain) were harvested and plated on PDA plates and fungal growth was enumerated. **, *P*<0.001; ***, *P*<0.0001; ****, *P*<0.00001; ns, not significant.

In the second series of RIT experiments we infected irradiated mice on day 3 post-irradiation with *R. oryzae* and treated them with RIT 24 h after infection. As the dose of 100 µCi proved to be more effective than 50 µCi of either antibody to melanin or to 1,3-beta-glucan, only a 100 µCi dose was used in these series. To establish whether unlabelled (‘cold’) antibodies have any effect on the fungal burden, cold antibody controls were used in addition to untreated mice and mice treated with radiolabelled control antibody MOPC21. Fig. 7 reveals that RIT with either 100 µCi ^177^Lu–DOTA-400–2 or 100 µCi ^177^Lu–DOTA–c8C3 resulted in a highly significant decrease in fungal burden in the brain, lungs, liver and spleen, but not the lungs, in comparison to controls in 100 µCi ^177^Lu–DOTA–MOPC21. In addition, cold c8C3 and cold 400–2 had minimal effect on the fungal burden of all harvested organs.

## Discussion

In the current study we investigated the possibility of using RIT targeting fungal pan-antigens 1,3-beta-glucan and melanin expressed by *R. oryzae* [20–22] for treating this infection in murine models. ELISA demonstrated that 400–2 mAb to 1,3-beta-glucan and c8C3 mAb to melanin were able to bind specifically to their respective antigens in both *R. oryzae* spores and hyphae, thus enabling the application of these mAbs in their radiolabelled form for *in vivo* experiments. Lower binding of 400–2 to hyphae than to the spores might be explained by the lower expression of 1,3-beta-glucan on the surface of the developing hyphae. The specificity of 400–2 and c8C3 mAbs was further confirmed during microSPECT/CT imaging of mice infected systemically with *R. oryzae*, which allowed us to proceed with RIT of infected mice.

Although the expression of 1,3-beta-glucan in *R. oryzae* is not exuberant, nevertheless its presence is documented in the literature [20, 21] and successful targeting of this antigen with RIT would imply that even fungi with relatively low levels of 1,3-beta-glucan expression could be targeted with RIT. Importantly, we have previously demonstrated successful killing *in vitro* and *in vivo* of *B. dermatitidis* [13], which is another human pathogenic fungus with low expression of 1,3-beta-glucan. The c8C3 mAb to melanin is a chimeric version of an original murine 8C3 mAb raised against the dimorphic fungal pathogen *Paracoccidioides brasiliensis* [23]. The 8C3 mAb cross-reacted with melanins from other pathogenic fungi, as well as with the synthetic and *S. officinalis* (cuttlefish) melanin [23], confirming it as a pan-melanin antibody. Likewise, mAbs raised against DHN or DOPA melanins from various fungi are capable of specific binding to various melanins, such as DHN melanin from *Aspergillus* spp., DOPA melanin from *C. neoformans,* synthetic DOPA melanin and *S. officinalis* melanin [24, 25]. Taken together, these data support our choice of 1,3-beta-glucan and melanin as pathogen-agnostic antigens for targeting with RIT.

In humans, rhinocerebral mucormycosis affects the sinuses and the brain; pulmonary mucormycosis leads to lung infection; cutaneoous mucormycosis involves skin infections; gastrointestinal mucormycosis causes nauseas, vomiting, abdominal pain and intestinal bleeding; and less is common renal mucormycosis [26]. In the murine model of systemic mucormycosis used in this work the fungus penetrates the blood–brain barrier (BBB) within 24 h post-infection [17], thus allowing us to simulate to some extent rhinocerebral infection in patients. Most likely, the infection of a murine brain compromises the BBB to some extent, as the radiolabelled mAbs to 1,3-beta-glucan and melanin were able to penetrate BBB and significantly reduce the fungal burden in the brain. In our previous work we observed that compromised BBB in mice with systemic cryptococcal infection allowed for a radiolabelled mAb to *C. neoformans* polysaccharide to enter the central nervous system (CNS) and decrease the cryptococcal burden in the brain to almost undetectable levels [27]. These results are significant, as fungal meningitis remains a serious cause of morbidity and mortality in patients with IFIs [28, 29]. Likewise, RIT was able to significantly reduce the fungal burden in lungs, spleen and liver, which are targets for *R. oryzae* infection. Interestingly, the reduction of the fungal burden in the organs in immunocompromised mice was more pronounced than the one in immunocompetent mice. This observation confirms the utility of the RIT approach in the setting of immunosuppression. The only organ where there was only a trend towards the reduction of the fungal burden with no statistical significance was the kidneys (Figs 5 and 7). The reasons behind this observation might be multiple, but the main one seems to be the exclusion of large molecules such as full size antibodies from the glomerular filtration. In future work, repeated administrations of RIT should be investigated for achieving better clearance of the infection from the kidneys.

One limitation of this study is that anti-fungals were not used as a comparator for RIT. However, in our previous RIT study treating *C. neoformans* infection in mice with organism-specific mAb [27], where amphotericin B was used as a comparator, even 14 days of treatment with amphotericin B produced minimal effect on the organs’ fungal burden. Such prolonged timeline is not compatible with the fast developing and later clearing infection in the current *R.oryzae* model [17]. In addition, anti-fungals are toxic even to immunocompetent mice, as shown in studies of various anti-fungals causing death of mice around day 24 [30, 31]. The clinical studies in immunocompromised patients also demonstrate that a short course of amphotericin B does not sterilize cerebrospinal fluid or blood and that the rate of sterilization correlates with survival [32].

The use of monoclonal antibodies targeting surface antigens as a new therapeutic strategy to treat fungal infections is currently gaining momentum [33, 34]. The distinct advantages of RIT over naked monocloncal antibodies and small molecule antifungals are: (1) its cytocidal nature, meaning that RIT does not merely abrogate a single cellular pathway but physically destroys targeted cells or cellular machinery; (2) it is less subject to drug resistance mechanisms [35]; (3) its efficacy is independent of the immune status of a host; (4) it has low toxicity in comparison to chemotherapy due to the specific tumour targeting. Our laboratory has demonstrated that micro-organism-specific mAbs are highly effective for the treatment of experimental fungal infections and other infections, such as those caused by bacteria and viruses (reviewed in [36]). It is worthwhile to note that the radionuclide used for RIT in this work was a beta-emitter, ^177^Lu, while all previous results on RIT targeting fungal pan-antigens were obtained with an alpha-emitter, bismuth-213 (^213^Bi) [12–14]. Taking into consideration worldwide challenges with the supply of alpha-emitting radionuclides, we made a decision to use the beta-emitter, ^177^Lu, which is readily available worldwide and has a relatively long physical half-life that permits cross-country transportation of radiopharmaceuticals, while ^177^Lu-labelled radiopharmaceuticals have been recently approved by the US Food and Drug Administration (FDA) and Health Canada for treatment of neuroendocrine tumours (Lutathera) and metastatic prostate cancer (Pluvicto).

Radioimmunoimaging and RIT in the field of infectious diseases in general and in fungal infections in particular is still much behind oncology [37–39]. Developing radioimmunoimaging for opportunistic fungal infections is very important because of the need to diagnose them in an expedited manner as the growth of fungal cultures obtained from patients might take weeks, which is often not feasible for critically ill patients. Very recently there have been some encouraging developments in pre-clinical and first-in-human positron emission tomography (PET) clinical imaging of *Aspergillus* lung infections in leukaemia patients targeting β−1,5-galactofuranose with a humanized mAb hJF5 [40].

## Conclusions

In conclusion, we have demonstrated in experimental murine mucormycosis that ^177^Lu-labelled mAbs to fungal pan-antigens 1,3-beta-glucan and melanin effectively decreased the fungal burden in major organs, including the brain. These results are encouraging because they show the effectiveness of pathogen-agnostic RIT in significantly decreasing fungal burden *in vivo*, while they can also potentially be applied to treat the broad range of invasive fungal infections that express pan-antigens 1,3-beta-glucan or melanin.

## Supplementary Data

Supplementary material 1
