# Supplementary material for: Radioimmunotherapy as a pathogen-agnostic treatment method for opportunistic mucormycosis infections
Source: Access Microbiol. 2023 Dec 6;5(12):000671.v4. doi: 10.1099/acmi.0.000671.v4 (PMC10765049; doi:10.1099/acmi.0.000671.v4)
Supplement: Supplementary material 1 [file acmi-5-671.v4-s001.pdf]

Supplementary Methods

Laminarin ELISA Protocol for Evaluating 400-2 antibody immunoreactivity

1.Buffers, Materials, Solutions and Reagents
1.1.Ninety-six-well Maxisorp plates (Nunc, Thermo Fisher Scientific)
1.2.25 µg/ml Laminarin in 0.1M bicarbonate buffer, pH 9.6
1.3.Mouse Monoclonal (1->3)-beta-glucan IgG, Kappa Light (400-2 Biosupplies), 1 mg/mL
1.4.Anti-mouse HRP secondary, 1:10,000 in PBST (Goat Anti-mouse IgG-HRP Conjugate, BioRad)
1.5. TMB Reagent (Thermo Scientific, bring to room temperature & prepare shortly before use)
1.6.0.1 M carbonate/bicarbonate buffer, pH 9.6
1.7.PBST (PBS with 0.05% Tween 20, pH 7.4)
1.8.3% BSA in PBST
1.9.1 M HCl stop solution

2. Immunoassay Procedure
2.1.Coat overnight at +4◦C with 5 µg laminarin in 0.1 M bicarbonate buffer at pH 9.6, 200 µL per well, columns 1-11
2.2.Wash plate 3x with 300 µL per well of PBST
2.3.Block with 200 µL/well of 3% BSA for 1 h at 37◦C
2.4.3x PBST wash
2.5.Pipette 300 µL of primary antibody (400-2) diluted to 1 µg/mL in the first well(s) column 1
2.6.Pipette 150 µL of PBST in subsequent wells columns 2-12
2.7.Serially dilute 1:2 across plate to column 11, leaving column 12 as the blank, by transferring 150 µL from column 1 to column 2, pipetting up and down 10 times, then transferring 150 µL from column 2 to column 3, etc. Repeat this pattern until column 11. Discard remaining 150 µL from column 11
2.8.Incubate at 37◦C for 2 hours
2.9. Perform 3x PBST wash
2.10. Add 100 µL/well of 1:10,000 in PBST Secondary anti-mouse HRP-Ab - incubate for 1 h at 37◦C
2.11. Perform 3x PBST wash
2.12. Develop plates for 6 mins at RT with 100 µL per well of TMB reagent
2.13. Add stop solution (1M HCl) and read the plate at 450 nm in a microplate reader.

Melanin ELISA: C8C3 + *Sepia officinalis* melanin Protocol

1.Buffers, Solutions and Reagents
1.1. Wash buffer: PBST (PBS + 0.05% Tween20 (TWEEN 20, pcode P13-79, Sigma-Aldrich, St. Louis, MO))
1.2. Samples Dilution buffer/Blocking buffer: 5% BSA in PBST
1.3. Coating buffer: PBS
1.4. Melanin from *Sepia officinalis* (cat# M2649, Sigma-Aldrich, St. Louis, MO)
1.5. Secondary Antibody: Peroxidase-conjugated anti-human IgG. 1:1000 dilution in Blocking buffer, prepare fresh immediately before use

1.6. TMB Microwell Peroxidase Substrate System (product code 50-76-00, KPL, Gaithersburg, MD): Bring to RT and prepare shortly before and on each day of use by mixing equal volumes of reagent A and reagent B
1.7. Stop solution: 1M HCl

2. Nunc Immobilizer Amino 96-well plate (cat# 436013, Thermo scientific, Rockford, IL)
Coating and Blocking
2.1. Prepare coating solution: add 60 mg of melanin to 12 ml of PBS to achieve 5mg/ml concentration.
2.2. Dispense 100 ul of 5mg/ml of coating solution to each well of Nunc Immobilizer Amino 96-well plate.
2.3. Incubate overnight at 45^oC (unsealed plates).
2.4. Wash 3 times with 300ul/well of Wash buffer, filling/ emptying the wells completely.
2.5. Dispense 200 ul of Blocking buffer into each well and incubate overnight at 4^oC.
2.6. Aspirate Blocking buffer, wash 3 times with 300 ul/well of Wash buffer

3. Immunoassay Procedure
3.1. Dilute testing antibody to a concentration of 100 ug/ml with Blocking/Dilution Buffer.
3.2. Dispense 100 ul/well of Blocking Buffer into columns 2-12
3.3. Dispense 200 ul of 100 ug/ml testing antibody in duplicates into column 1.
3.4. Serially dilute 1:2 across plate to column 11, leaving column 12 as the blank, by transferring 100 ul from column 1 to column 2, pipetting up and down 10 times, then transferring 100ul from column 2 to column 3. Repeat this pattern until column 11. Discard remaining 100 ul from column 11.
3.5. Incubate plate at 37^oC for 1.5 hr
3.6. Aspirate the testing antibody; wash 3 times with 300 ul/well of Wash buffer
3.7. Dilute secondary Peroxidase-conjugated Anti-human IgG to 1:1000 with blocking/Dilution Buffer.
3.8. Dispense 100ul/well of diluted secondary Peroxidase-conjugated Anti-human IgG into all wells.
3.9. Incubate the plate at 37^oC for 1.5 hr
3.10. Aspirate HRP Anti-Human IgG, wash 3 times with 300 ul/well of Wash buffer
3.11. Immediately before use, mix TMB substrate and TMB solution together.
3.12. Dispense 100 ul/well of TMB into all wells. Protect from light. Incubate plate at RT for 10 min.
3.13. Dispense Stop Solution and read plate at 450 nm

ELISA Protocol for evaluating immunoreactivity of 400-2 and C8C3 antibodies towards *Rhizopus oryzae* spores or hyphae

1.Buffers, Materials, Solutions and Reagents
1.1. Wash buffer: PBST (PBS + 0.05% Tween20 (TWEEN 20, pcode P13-79, Sigma-Aldrich, St. Louis, MO))
1.2. Samples Dilution buffer/Blocking buffer: 3% BSA
1.3. 8% paraformaldehyde
1.4. Rhizopus oryzae spores: $4x10^7$ spores/ml

1.5. Rhizopus oryzae hyphae: 60 µg of hyphae in 10 ml/PBS (100 µl/48 wells)
1.6. Secondary Antibodies: Peroxidase-conjugated anti-human IgG (Anti-human-HRP Conjugate, BioRad), 1:1000 dilution in Blocking buffer for C8C3 and Anti-mouse HRP secondary, 1:10,000 in PBST (Goat Anti-mouse IgG-HRP Conjugate, BioRad) for 400-2
1.7. TMB Microwell Peroxidase Substrate System (product code 50-76-00, KPL, Gaithersburg, MD)
1.8. Stop solution: 1M HCl
1.9. Nunc Immobilizer Amino 96-well plate (cat# 436013, Thermo scientific, Rockford, IL)
Coating and Blocking

2. Fix Spores or Hyphae to the Plate

2.1. Add aliquot of 25µl (10^6 spores) of spore suspension to each well; or add an aliquot of 100 µl of hyphae suspension (0.6 µg) to each well (columns 1-11)
2.2. Add 100 µL of 8% paraformaldehyde solution to each well to fix and crosslink the spores or hyphae to the microplate.
2.3. Incubate at 45°C for 15 minutes
2.4. Wash 3 times with 300ul/well of Wash buffer (PBST), filling/ emptying the wells completely.
2.5. Dispense 200ul of Blocking buffer (3%BSA) into each well and incubate for 30 minutes at 37° C.
2.6. Aspirate Blocking buffer, wash 3 times with 300ul/well of Wash buffer

3. Immunoassay Procedure

3.1. Pipette 300 µl of the primary antibody (400-2 or C8C3) diluted to 1 µg/mL in the first well(s) column 1.
3.2. Pipette 150 µL of PBST in subsequent wells columns 2-12
3.4. Serially dilute 1:2 across plate to column 11, leaving column 12 as the blank, by transferring 150 µl from column 1 to column 2, pipetting up and down 10 times, then transferring 150 µl from column 2 to column 3. Repeat this pattern until column 11. Discard remaining 150 µl from column 11.
3.5 Incubate at 37◦C for 1 hour
3.6 Perform 3x PBST wash
3.7 Add 100 µL/well in PBST of either anti-mouse (for 400-2 primary antibody) or anti-human (for C8C3 primary antibody) secondary HRP-Ab - incubate for 1 h at 37◦C
3.8 Perform 3x PBST wash
3.9 Develop plates for 6 mins at RT with 100 µL per well of TMB reagent
3.10 Add stop solution (1M HCl) and read the plate at 450 nm in a microplate reader.
